# Association and Interaction Effect of *BHMT* Gene Polymorphisms and Maternal Dietary Habits with Ventricular Septal Defect in Offspring

**DOI:** 10.3390/nu14153094

**Published:** 2022-07-28

**Authors:** Manjun Luo, Tingting Wang, Peng Huang, Senmao Zhang, Xinli Song, Mengting Sun, Yiping Liu, Jianhui Wei, Jing Shu, Taowei Zhong, Qian Chen, Ping Zhu, Jiabi Qin

**Affiliations:** 1 Department of Epidemiology and Health Statistics, Xiangya School of Public Health, Central South University, Changsha 410078, China; lmj1990dlh@163.com (M.L.); zsmhncs@163.com (S.Z.); xinlisong@foxmail.com (X.S.); sunmtbeloved@163.com (M.S.); 18843113354@163.com (Y.L.); weijihi@163.com (J.W.); sj1234511@163.com (J.S.); taoweizhong98@163.com (T.Z.); chenqian07172022@163.com (Q.C.); 2 National Health Committee Key Laboratory of Birth Defect for Research and Prevention, Hunan Provincial Maternal and Child Health Care Hospital, Changsha 410028, China; 3 Department of Cardiothoracic Surgery, Hunan Children’s Hospital, Changsha 410007, China; hpeng1979@163.com; 4 Guangdong Cardiovascular Institute, Guangdong Provincial People’s Hospital, Guangdong Academy of Medical Sciences, Guangzhou 510080, China; 5 Hunan Provincial Key Laboratory of Clinical Epidemiology, Changsha 410078, China; 6 Guangdong Provincial Key Laboratory of Pathogenesis, Targeted Prevention and Treatment of Heart Disease, Guangzhou 510640, China

**Keywords:** ventricular septal defects, *BHMT* gene plolymorphisms, dietary habits, interaction effects

## Abstract

This study attempted to learn the association between maternal betaine-homocysteine methyltransferase (*BHMT*) gene polymorphisms, maternal dietary habits, and their interactions with the risk of ventricular septal defects (VSD) in offspring. A total of 426 mothers of VSD children and 740 control mothers were included in the study. Logistic regression was used to evaluate the level of associations and interaction effects. Our study suggested that mothers reporting excessive intake of smoked foods (aOR = 2.44, 95%CI: 1.89–3.13), barbecued foods (aOR = 1.86, 95%CI: 1.39–2.48), fried foods (aOR = 1.93, 95%CI: 1.51–2.46), and pickled vegetables (aOR = 2.50, 95%CI: 1.92–3.25) were at a significantly higher risk of VSD in offspring, instead, mothers reporting regular intake of fresh fruits (aOR = 0.47, 95%CI: 0.36–0.62), fish and shrimp (aOR = 0.35, 95%CI: 0.28–0.44), fresh eggs, (aOR = 0.56, 95%CI: 0.45–0.71), beans (aOR = 0.68, 95%CI: 0.56–0.83), and milk products (aOR = 0.67, 95%CI: 0.56–0.80) were at a lower risk of VSD in offspring. In addition, maternal *BHMT* gene polymorphisms at rs1316753 (CG vs. CC: aOR = 2.01, 95%CI: 1.43–2.83) and rs1915706 (CT vs. TT: (aOR = 1.81, 95%CI: 1.33–2.46) were significantly associated with increased risk of VSD in offspring. Furthermore, a significant interaction between *BHMT* polymorphisms and maternal bean intake was identified in the study. In conclusion, Maternal *BHMT* polymorphisms at rs1316753 and rs1915706, dietary habits as well as their interaction were observed to be significantly associated with the risk of VSD in offspring.

## 1. Introduction

Congenital heart disease (CHD) is typically defined as a gross structural abnormality of the heart and/or great vessels that is present at birth [1,2]. It has been reported that the birth prevalence of CHD has increased significantly since the 1930s and reached a maximum of over 9 per 1000 live births since 1995 [1,3]. Ventricular septal defect (VSD) has been recognized as the most common congenital cardiac malformation and accounts for roughly 30–40% of all cardiac anomalies [1,4]. Over the past decades, considerable inherited causes and noninherited modifiable factors have been implicated in the development of CHD and its subgroups [5,6,7,8]. Recently, there has been a consensus that genetic factors and environmental factors interact in the etiology of most nonsyndromal forms of CHD [9,10], naturally including VSD.

A recent review showed strong evidence that oral prenatal fortification and supplementation dosing of folic acid (FA) can prevent the incidence of VSD and atrial septal defect (ASD) [11]. Women with a diverse diet during pregnancy (dietary diversity score, DDS ≥5) had lower risks of having fetuses with total CHD and VSD [12]. Furthermore, the dietary intake of vitamins and minerals was found to be associated with a reduced risk of CHD in offspring, including B-vitamin, vitamin D, zinc, and selenium [13,14,15]. Since different nutrients interact with one another in many metabolic pathways, it seems that the association would not remain constant when various nutrients coexist in the same food. In addition, the dietary pattern differs a lot owing to the discrepancy in economics, geographical environment, social culture, race, and so on. Therefore, the first concern we would care to discuss is the association between maternal dietary habits and VSD in offspring.

**Figure 1:**
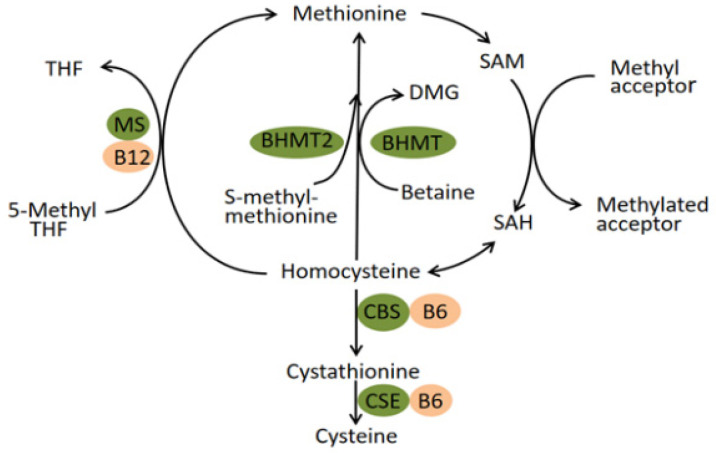
Pathways of homocysteine metabolism. Abbreviation: BHMT Betaine-homocysteine S-methyltransferase; MS methionine synthase; CBS cystathionine β-synthase; CSE cystathionine-γ-lyase; THF tetrahydrofolate; DMG dimethylglycine; SAM S-adenosylmethionine; SAH S-adenosylhomocysteine.

The human betaine-homocysteine methyltransferase (BHMT) gene maps to 5q13.1–q15, spans about 20 kilobases of DNA and contains eight exons and seven introns [16,17]. The enzyme it encodes, betaine-homocysteine methyltransferase, catalyzes the transfer of a methyl group from betaine to homocysteine (Hcy), forming dimethylglycine and methionine. Generally, the homeostasis of plasma homocysteine benefits from the transulfuration pathway involving cystathionine β synthase (CBS) and the remethylation pathway involving BHMT, BHMT2, and methionine synthase (MS) (Figure 1) [18]. In the latter pathway, the catalytic activity of BHMT2 is absolutely diet-dependent since its substrate, S-methylmethionine, can only be biosynthesized by various plants mainly belonging to the Brassicaceae family rather than mammals [19,20]. Experimental research conducted in mice suggested that BHMT is a predominant enzyme for the elimination of Hcy while the MS has little excess capacity to methylate the Hcy [18]. Therefore, the remethylation reaction catalyzed by BHMT seems to play a vital role in preventing the toxic accumulation of Hcy. In fact, BHMT catalyzes up to 50% of homocysteine metabolism in the human liver, where the enzyme is highly expressed [21,22]. The latest literature revealed that elevated Hcy concentrations acted as a risk factor for multiple congenital anomalies in human production, mainly comprising neural tube defects (NTD), orofacial clefts, and CHD [23,24,25]. The discovery has been generally accepted that the 677 C→T mutation in the methylenetetrahydrofolate reductase (MTHFR) gene contributed to elevated tHcy and is a genetic risk factor for diseases associated with hyperhomocysteinaemia [26]. Moreover, this mutation has been applied to antenatal screening for pregnant women in China. The thought naturally emerged that polymorphisms of the BHMT gene exist that reduce BHMT activity and increase plasma Hcy levels and thus increase malformation risk. In fact, research has been dedicated to exploring the association between BHMT gene polymorphisms and CHD, but with fixed results and little involving subgroups of CHD [27,28,29,30]. In this study, we focused on the largest subcategory of CHD, namely, VSD, to detect its association with polymorphisms of the maternal BHMT gene.

In addition, betaine, the substrate of BHMT, can be either obtained from food resources or produced from choline endogenously [31]. Likewise, choline can also be produced endogenously via the hepatic phosphatidylethanolamine N-methyltransferase (PEMT) pathway. However, most people must consume this nutrient exogenously to prevent deficiency [32]. Therefore, the BHMT activity, to a certain degree, is diet dependent. Animal studies did observe that pane of nutrition or the supply of some nutrients, including choline and methionine, can alter BHMT activity [33,34,35]. In addition, it has been reported that women with a high intake of one-carbon cofactors had a lower risk of congenital anomalies in offspring, such as the neural tube defect (NTD) and perimembranous ventricular septal defect (VSD_pm_) [36,37]. Overall, these valuable clues were collected to put forward a reasonable hypothesis that BHMT gene polymorphisms may interact with maternal dietary habits on congenital anomalies.

In this study, we determined VSD, the most common subgroup in CHD, as the interested outcome variable, which is relatively more sensitive to maternal nutrient intake. A hospital-based case-control study was carried out in an attempt to learn the following questions: a. the association of maternal dietary habits with risk of VSD in offspring; b. the association of polymorphisms of maternal BHMT gene with risk of VSD in offspring; c. the interaction between BHMT genetic variants and maternal dietary habits on VSD.

## 2. Materials and Methods

### 2.1. Design and Participants

This is a hospital-based case-control study that started in February 2018 and was over in March 2020. The cases and controls came from different departments in the same hospital, Hunan children’s hospital, which is famous partly for its sophisticated diagnosis and treatment techniques for CHD within the province. Considering the characteristics of the relatively low incidence of VSD compared with other chronic diseases, a convenient sampling method was used in the recruitment of the cases. VSD children, verified by both doppler echocardiography and surgery, were consecutively recruited from the Department of Cardiothoracic Surgery. Children in the control, free of any congenital malformations, were randomly selected from the Department of Child Healthcare. It is worth noting that cases only included VSD children that may or may not be diagnosed with other congenital heart diseases; those coexisting with any other extra-cardiac malformations were excluded from the study. Additionally, informed consent was obtained from all of the participants, and the possible consequences of the study were explained. The exclusion criterions mainly included: minority mothers, mothers conceiving children through in vitro fertilization or other conception methods, adoptive mothers or stepmothers, and mothers suffering from mental disorders or any other physical diseases so that this did not hinder the provision of accurate exposure information and blood samples. Finally, a total of 426 mothers of VSD children and 740 control mothers were included in the study.

The protocol of this study was in accordance with the guidelines of the 1964 Helsinki Declaration, and the Ethics Committee of Xiangya School of Public Health, Central South University, officially approved this study in January 2018. (no. XYGW-2018-36).

### 2.2. Information Collection

The outcome we focused on in the study was VSD in offspring, which was diagnosed by professional physicians via both doppler echocardiography and surgery. The interested exposures were maternal dietary habits in early pregnancy, which were collected from a self-designed food frequency questionnaire. We consulted The Dietary Guidelines for Chinese Residents and went deep into the local food culture to develop the questionnaire. Eleven main categories were determined, involving smoked foods, barbecued foods, fried foods, pickled vegetables, fresh vegetables, fresh fruits, fresh meat, fish and shrimp, fresh eggs, beans, and milk products. Each category was provided with three choices: a. hardly (less than or equal to two times per week); b. sometimes (three to five times per week); c. often (more than or equal to six times per week). The questionnaire was pre-investigated using eligible mothers (test–retest reliability: r = 0.826; internal consistency: α = 0.769).

In addition, we also collected various pieces of maternal information that might influence the outcomes of their offspring, mainly including the child-bearing age (<35 years or ≥35 years), pre-pregnancy BMI (calculated with their pre-pregnancy height and weight, <18.5, 18.5–23.9, 24–26.9, or ≥27), education level (less than primary or primary, junior high school, high school or technical secondary school, college or above), consanguineous marriages (yes or no), gestational diabetes mellitus (yes or no), gestational hypertension (yes or no), abnormal pregnancy history before this pregnancy (yes or no), congenital malformations in family members (yes or no), exposure of environmental pollutants (yes or no), antibiotic use in early pregnancy (yes or no), tobacco exposure in early pregnancy (yes or no), alcohol exposure in early pregnancy (yes or no), and periconceptional folate use (yes or no).

An epidemiological survey was conducted by well-trained investigators when participants were waiting for their operation arrangements in the wards or medical check-ups in the Department of Child Health. In China, every expectant mother has a personal Maternal and Child Health Manual, which provides their sociodemographic information, the results of regular medical check-ups, and necessary exposure information. So, in the course of the investigation, we consulted the participants’ manual to further confirm the abovementioned information obtained from face-to-face interviews, which enabled us to reduce recall bias to a certain extent.

### 2.3. Sample Collection and Genotyping

Five milliliters of peripheral venous blood were collected from every single participant after the face-to-face interview. All of the obtained blood samples would be brought back to the laboratory at low temperatures (≤4 °C) within twelve hours and then divided into two layers using a high-speed centrifuge: the blood cell layer and the plasma layer. Both were stored in an ultra-low-temperature freezer until genotyping. The DNA was extracted from the blood cell samples with the QIAamp DNA Blood Mini Kit (Qiagen, Valencia, CA, USA). Genotyping was performed by matrix-assisted laser desorption and ionization time-of-flight mass spectrometry MassARRAY system (Agena iPLEX assay, San Diego, CA, USA). The laboratory technicians who performed SNP detection and recorded the genotype data were blind to whether each sample was from the cases or controls, thereby reducing selection bias to some extent.

Before genotyping, we consulted the NCBI and HapMap databases to determine the major SNP sites of the *BHMT* gene and simultaneously excluded the SNPs whose minor allele frequencies (MAF) were less than 10%. Furthermore, we imposed a minimum SNP genotyping call rate at the level of 50%, which was applied to ensure the data integrity of the individual’s genotypes. Moreover, the success rates for the SNPlex assays were >94% for 2 SNPs, from 90 to 94% for 2 SNPs. Finally, these genetic loci (rs3733890, rs1316753, rs567754, and rs1915706) were selected as candidate loci for this study.

### 2.4. Statistical Analysis

The data for the qualitative variables were expressed as absolute numbers (percentages). The chi-square test was used to assess the differences in qualitative variables across groups. The Hardy–Weinberg equilibrium (HWE) test was used to compare the differences in genotype distribution frequency in the control group (significance level at *p* < 0.01). We utilized a logistic regression model to detect whether the association between maternal dietary habits in early pregnancy, *BHMT* gene polymorphisms, and VSD in offspring existed and the level of the association. Both univariate and multivariate logistic regressions were adopted; the crude odds ratio (cOR) and its 95% confidential intervals (CI) were calculated by the former one without any adjustment; the adjusted odds ratio (aOR) and its 95% confidential intervals (CI) were calculated by the latter one, which adjusted for the significant confounders found using the chi-square test. For the significant SNPs and maternal dietary habits, these originally ternary variables were converted into binary variables. We then introduced all of the potential confounders, genetic factors, environmental factors, and their multiplicative interaction term into the same logistic regression model to determine the presence or absence of gene–environment interaction and assess its significance. When it comes to multiple hypothesis testing, the false discovery rate (FDR) based on the Benjamini–Hochberg method was used to correct for bias. A false discovery rate P value (FDR_P) of <0.1 was considered to be statistically significant. The calculation of FDR_P was completed using R software (version 4.1.3, stats package). Basic analyses were performed using SPSS 26.0 software (SPSS Inc., Chicago, IL, USA). The statistically significant results were those with the two-sided *p*-value < 0.05, except where otherwise specified.

## 3. Results

### 3.1. Comparison of Maternal Baseline Characteristics

In the study, we recruited a total of 426 mothers of VSD children for cases and 740 mothers of non-congenital malformation children for controls. The selection of participants conformed strictly to the pre-made inclusion and exclusion criteria. The median (inter-quartile range) age of the children the in cases and controls was 8.4 (5.7) months and 7.8 (4.3) months, respectively. The comparisons of the maternal baseline characteristics between cases and controls are summarized in Table 1. There were statistically significant differences between the two groups in the following factors: pre-pregnancy BMI, education level, consanguineous marriages, gestational diabetes mellitus, gestational hypertension, abnormal pregnancy history before this pregnancy, congenital malformations in family members, exposure to environmental pollutants, antibiotic use in early pregnancy, tobacco exposure in early pregnancy, alcohol exposure in early pregnancy, and periconceptional folate use (all *p* values < 0.05). These abovementioned factors would be adjusted as confounders when evaluating the association of maternal dietary habits, SNPs of the *BHMT* gene, and their interactions with VSD in offspring.

### 3.2. Maternal Dietary Habits and the Risk of VSD in Offspring

The association of maternal dietary intake in early pregnancy with the risk of VSD in offspring is shown in Table 2. Both univariate and multivariate logistic regression indicated that smoked foods, barbecued foods, fried foods, pickled vegetables, fresh fruits, fish and shrimp, fresh eggs, beans, and milk products were significantly associated with the risk of VSD in offspring. Specifically, children were predisposed to VSD when their mothers reported excessive intake of smoked foods (aOR = 2.44, 95%CI: 1.89–3.13), barbecued foods (aOR = 1.86, 95%CI: 1.39–2.48), fried foods (aOR = 1.93, 95%CI: 1.51–2.46), and pickled vegetables (aOR = 2.50, 95%CI: 1.92–3.25). Instead, a significantly decreased risk of VSD was observed in children whose mothers reported regular intake of fresh fruits (aOR = 0.47, 95%CI: 0.36–0.62), fish and shrimp (aOR = 0.35, 95%CI: 0.28–0.44), fresh eggs, (aOR = 0.56, 95%CI: 0.45–0.71), beans (aOR = 0.68, 95%CI: 0.56–0.83), and milk products (aOR = 0.67, 95%CI: 0.56–0.80).

### 3.3. Maternal BHMT Gene Polymorphisms and the Risk of VSD in Offspring

Table 3 displays the genotypic distribution of four SNPs between two groups and the results of the HWE test in the controls. All of the SNPs were in accordance with the Hardy–Weinberg equilibrium (all of the *p* values were <0.05), indicating that the sample was qualified for good group representativeness.

The association of maternal *BHMT* gene polymorphisms with the risk of VSD in offspring based on logistic regression analysis was summarized in Table 4. After adjusting for potential confounders, statistically significant associations were found between the polymorphisms of the *BHMT* gene at rs1316753, rs1915706, and VSD in offspring. For rs1316753, mothers carrying the CG genotype (aOR = 2.01, 95%CI: 1.43–2.83) were at a significantly higher risk of VSD in offspring compared with those who had the CC genotype. In addition, the dominant model (aOR = 1.88, 95%CI: 1.36–2.61) and the additive model (aOR = 1.30, 95%CI: 1.06–1.60) of rs1316753 were also observed to be significantly associated with increased risk of VSD in offspring. For rs1915706, compared to the TT genotype, mothers with the CT genotype (aOR = 1.81, 95%CI: 1.33–2.46) were more likely to have VSD children. Additionally, the dominant model (aOR = 1.84, 95%CI: 1.37–2.48) and the additive model (aOR = 1.61, 95%CI: 127–2.05) of rs1915706 were significantly associated with an increased risk of VSD in offspring.

### 3.4. Interaction of the Polymorphisms of BHMT Gene and Maternal Dietary Habits on the Risk of VSD in Offspring

**Figure 2:**
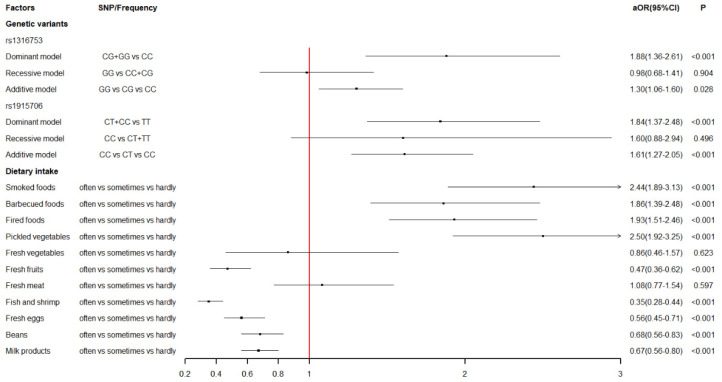
The level of association between genetic variants of *BHMT* gene, maternal dietary intake and VSD in offspring.

Figure 2 shows the level of association between genetic variants of the *BHMT* gene, maternal dietary intake, and VSD in offspring. The interaction of *BHMT* gene polymorphisms and maternal dietary habits in early pregnancy based on multivariate logistic regression analysis is displayed in Table 5. For rs1316753, statistically significant interactions were observed between the variant genotypes (CG + GG) and excessive intake of pickled vegetables (aOR = 0.48, 95%CI: 0.24–0.95) and beans (aOR = 0.40, 95%CI: 0.17–0.95). Nevertheless, this significance vanished from the multiple test corrections based on the Benjamini–Hochberg method (both FDR-*p* values > 0.1). As for rs1915706, there were significant interactions between the variant genotypes (CT + CC) and a regular intake of beans (aOR = 0.33, 95%CI: 0.15–0.73, FDR-*p* = 0.035).

**Figure 3:**
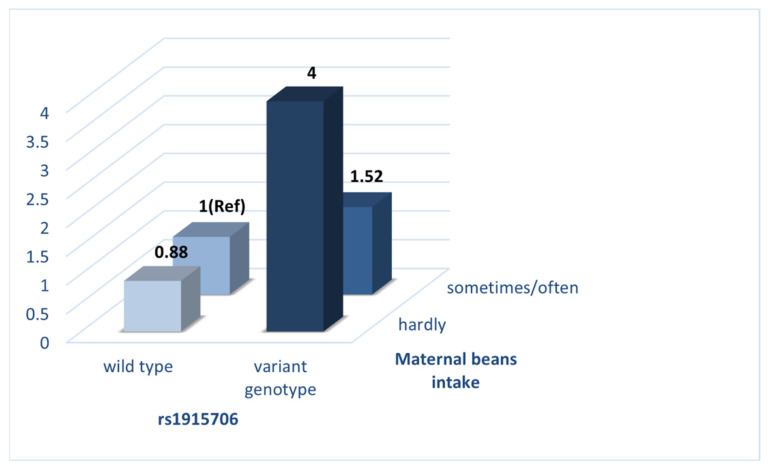
Interaction of rs1915706 and maternal beans intake on the risk of VSD in offspring.

The crossover analysis was conducted to further elucidate the interaction between *BHMT* gene polymorphisms at rs1915706 and maternal bean intake on the risk of VSD in offspring (Table 6 and Figure 3). Mothers who had the wild genotype (TT) and meanwhile reported regular intake of beans in early pregnancy were seen as the reference group. After adjustment for potential confounders detected in Table 1, mothers who had the variant genotypes (CT + CC) and meanwhile reported regular intake of beans (aOR = 1.52, 95%CI: 1.09–2.11) and a small intake of beans (aOR = 4.00, 95%CI: 2.17–7.40) were at a significantly higher risk of VSD in offspring compared with those who were in the reference group.

## 4. Discussion

Research on the causes of CHD has made great strides, and more than 400 genes and important environmental factors have been determined to have substantial evidence in relation to the risk of developing CHD and its subgroups [5,6,7,8]. However, the conclusion can be easily drawn from the other hand that a single genetic or environmental factor may impose minimal effects on CHD. Moreover, the interaction of the two factors cannot be overlooked in the occurrence and development of CHD and its subgroups. In this study, we attempted to achieve an insight into the etiology of VSD, the most common subtype of CHD. The main purpose was to decide whether the association and interaction effect of BHMT gene polymorphisms and maternal dietary habits with VSD exists, which is conducive to the achievement of molecular genetic diagnostics and provide diet instruction to expectant mothers in early pregnancy. 

Firstly, we explored the association between maternal dietary intake during early pregnancy and the risk of VSD in offspring. The results sent two messages. On the one hand, mothers who reported excessive intake of smoked foods (aOR = 2.44), barbecued foods (aOR = 1.86), fried foods (aOR = 1.93), and pickled vegetables (aOR = 2.50) were more likely to have a VSD-affected child. On the other hand, mothers with an excessive intake of fresh fruits (aOR = 0.47), fish and shrimp (aOR = 0.35), fresh eggs (aOR = 0.56), beans (aOR = 0.68), and milk products (aOR = 0.67) were less likely to have a VSD-affected child. Generally, various harmful chemicals can be generated from improper food processing, and most of them are teratogens and carcinogens, such as nitrites, acrylamide (ACR), polycyclic aromatic hydrocarbons (PAH), and so on. Pickled vegetables have a wide range of nitrite and nitrate contents. Gravidas, who have an excessive intake of pickles, may suffer hypoxemia because increased nitrite can react with hemoglobin, rendering it incapable of carrying oxygen [38]. A recent experimental study established a rodent model and reported that hypoxia was able to cause numerous abnormalities in cardiomyocyte gene expression, the electrophysiologic substrate of the heart, and contractile function, thus delaying cardiac maturation [39]. ACR, identified in food in 2002, is mainly formed via the Maillard reaction, whereby a carbonyl compound reacts with the amino group of asparagine processed at high temperatures (>120 °C, such as barbecuing, frying, and baking) [40,41]. Although no direct evidence manifested its relation to heart defects, a number of animal studies have shown strong neurotoxic, genotoxic, carcinogenic, mutagenic, and teratogenic effects [42]. Food is readily contaminated by PAH during the smoking process involving the combustion of fuel. A recent study reported that greater maternal levels of PAH exposure during pregnancy might be associated with an elevated prevalence of fetal CHD [43]. Moreover, prior experimental research provided strong evidence that PAH exposure can result in abnormal heart looping, an enlarged ventricle with a thinner ventricular wall, and even developmental cardiac defects [44,45]. The protective foods detected in the study, such as fruits, fish and shrimp, eggs, beans, and milk products, are common foods on tables. Furthermore, they are packed with numerous nutrients, such as high-quality protein, vitamins, minerals, and so on, which have been extensively accepted to play a vital role in maintaining the health of gravidas and fetuses. 

Moreover, we determined the association between maternal BHMT gene polymorphisms and the risk of VSD in offspring. Four SNPs (rs3733890, rs1316753, rs567754, and rs1915706) were considered in this study, and two SNPs (rs1316753 and rs1915706) were for the first time found to have a statistically significant association with VSD. To date, the BHMT gene remained relatively little studied compared with other folate- and homocysteine-metabolizing genes. The results in our study were only partly in accordance with prior studies. The rs3733890 polymorphism is a well-studied exon of the BHMT gene and undergoes a G-to-A change at nucleotide 716, leading to an arginine-to-glutamine substitution at amino acid 239. Its association with congenital defects has been widely explored but with contradictory results [27,46,47,48]. In the present study, we did not detect its significance in the occurrence of VSD. Rs567754 is an intronic variant of the BHMT gene, and neither previous data nor our data revealed an association with CHD or VSD in offspring [27]. The interesting thing is that two other SNPs, rs1316753 and rs1915706, were observed to be statistically associated with a remarkably-increased risk of VSD in offspring. To the best of our knowledge, experimental or epidemiological research involving these two polymorphisms remains a nearly unworked area, meaning that their potential functional effects on BHMT are largely unknown. Qiping Feng et al. performed a genotype–phenotype correlation analysis on betaine-homocysteine methyltransferase and found that three introns (rs41272270, rs6875201, and rs7700790) and an intergenic variant (rs16876512) were significantly correlated with both BHMT activity and protein levels [22]. Although this convincing research did not cover the two significant SNPs in our study, the analogy seems to be reasonable that the two intergenic variants, rs1316753 and rs1915706, are also capable of producing potential effects on the expression of the BHMT gene and subsequently influencing plasma hcy concentrations. The correlation between maternal hyperhomocysteinemia and CHD has been extensively studied and reviewed [25,49,50]. Meanwhile, hcy-induced CHDs, such as the transposition of the great arteries (TGA), single ventricle defects (SVD), and VSD, have been found in embryos of different species (mice and chicken) [51]. Therefore, the statistical association between maternal BHMT polymorphisms and VSD in offspring might be explained by the pathway from BHMT activity to elevated hcy levels to multiple congenital anomalies. Nonetheless, more related research is encouraged to provide clearer evidence, thus elucidating the potential mechanism.

Lastly, we also analyzed the gene–environment interaction and observed a significant interaction between genetic variants of the BHMT gene at rs1915706 and maternal bean intake on the risk of VSD in offspring. The expectant mothers who had the variant genotypes (CT + CC) and meanwhile reported a small intake of beans were at a significantly higher risk of VSD in offspring (aOR = 4.00) compared with those with the wild genotype (TT) and reported having a regular intake of beans in early pregnancy. Beans are an excellent source of zinc, choline, and multiple B vitamins (such as folate, thiamin, niacin, riboflavin, and pyridoxine) [52,53]. Notably, BHMT is a zinc-dependent cytosolic enzyme, and its substrate, betaine, is partly derived from dietary choline [31,54]. In addition, a stronger risk reduction in CHD has been found in the maternal folate + B-vitamin supplementation group compared with the maternal folate supplementation group, both setting the non-users as the reference group [14]. Concerning whether a single nutrient can exist in various foods, we speculated that the deficiency of diverse nutrients coexisting in beans coincided with a genetic variant that contributes to the occurrence of VSD. This speculation seemed plausible since similar research had been conducted not long ago. Hartmut Cuny et al. demonstrated that when dietary undersupply during pregnancy was combined with a maternal heterozygous variant in Haao, a gene of the nicotinamide adenine dinucleotide (NAD) synthesis pathway, the incidence of embryo loss and malformations was significantly higher [55]. This is a classical experimental study forcibly indicating a gene-diet interaction. As Gibson G and Berger K commented, the discovery of such interaction suggests that the close monitoring of nutrition in at-risk carrier mothers would be the type of personalized and predictive intervention that advocates of genomic health call for [56]. Regardless, what we found in our study necessitates more convincing experimental research and crowd investigation to confirm it repeatedly.

Furthermore, several limitations in our study should be acknowledged. Firstly, although we perfected the study design and executed it strictly during the whole process as far as possible, the association found in this study, an observational case-control study, was relatively limited compared to a cohort study or an experimental study. So, in other words, well-designed prospective cohorts or reasonable experimental research are needed to validate our findings further. Secondly, the information on food frequency and related exposure in the questionnaire were obtained through retrospective investigation; we cannot rule out the possible limitation of recall bias. Thirdly, this is a hospital-based case-control study, and all of the cases came from the same department in a hospital; though its representativeness in the province for sophisticated diagnosis and treatment techniques, the selection bias still cannot be ignored. Fourthly, several potential confounders were determined and adjusted in the study, but there undoubtedly are other confounding covariates that might also influence the outcomes, such as common genetic polymorphisms and some nutritional biomarkers. The findings would be more convincing if taking these factors into consideration. Last but not least, maternal hcy concentration was not available in our research, which excludes the possibility of verifying the potential explanation that genetic variants of the BHMT gene may cause VSD by elevating maternal hcy levels.

## 5. Conclusions

In this hospital-based case-control study, statistically significant associations were found between the polymorphisms of the *BHMT* gene at rs1316753, rs1915706, and VSD in offspring. Maternal dietary habits were also observed to have a significant impact on the occurrence and development of VSD. A significant interaction between *BHMT* polymorphisms and maternal bean intake was identified in the study. Concerning the limitations of our study, more convincing crowd investigation and experimental research are necessary to repeatedly verify the findings and further elucidate the potential mechanism.

## Figures and Tables

**Table 1:** Comparison of maternal baseline characteristics in cases and controls.

Baseline Characteristics	Control Group	Case Group	*χ* ^2^	*p*
	(n = 740)	(n = 426)		
Child-bearing age (years)			0.912	0.340
<35	635(85.8%)	374(87.8%)		
≥35	105(14.2%)	52(12.2%)		
Pre-pregnancy BMI ^a^				
<18.5	192(25.9%)	77(18.1%)	11.810	**0.008**
18.5–23.9	406(54.9%)	274(64.3%)		
24–26.9	91(12.3%)	47(11.0%)		
≥27	51(6.9%)	28(6.6%)		
Education level			187.573	**<0.001**
Less than primary or primary	9(1.2%)	43(10.1%)		
Junior high school	144(19.5%)	195(45.8%)		
High school or Technical secondary school	246(33.2%)	123(28.9%)		
College or above	341(46.1%)	65(15.3%)		
Consanguineous marriages			13.989	**<0.001**
No	737(99.6%)	413(96.9%)		
Yes	3(0.4%)	13(3.1%)		
Gestational diabetes mellitus			34.302	<0.001
No	717(96.9%)	376(88.3%)		
Yes	23(3.1%)	50(11.7%)		
Gestational hypertension			23.594	**<0.001**
No	723(97.7%)	390(91.5%)		
Yes	17(2.3%)	36(8.5%)		
Abnormal pregnancy history pregnancy			9.363	**0.002**
No	411(55.5%)	197(46.2%)		
Yes	329(44.5%)	229(53.8%)		
Congenital malformations in family members			19.837	**<0.001**
No	733(99.1%)	404(94.8%)		
Yes	7(0.9%)	22(5.2%)		
Exposure to environmental pollutants			43.687	**<0.001**
No	687(92.8%)	340(79.8%)		
Yes	53(7.2%)	86(20.2%)		
Antibiotic use in early pregnancy			7.234	**0.007**
No	729(98.5%)	409(96.0%)		
Yes	11(1.5%)	17(4.0%)		
Tobacco exposure in early pregnancy			78.692	**<0.001**
No	602(81.4%)	244(57.3%)		
Yes	138(18.6%)	182(42.7%)		
Alcohol exposure in early pregnancy			9.461	**0.002**
No	712(96.2%)	392(92.0%)		
Yes	28(3.8%)	34(8.0%)		
Periconceptional folate use			7.026	**0.008**
Yes	687(92.8%)	376(88.3%)		
No	53(7.2%)	50(11.7%)		

Abbreviations: BMI body mass index. ^a^ Classification according to Chinese standard for obesity BMI.

**Table 2:** Maternal dietary habits and the risk of VSD in offspring.

Maternal Dietary Habits	Control Group	Case Group	Univariate Logistic Regression		Multivariable Logistic Regression ^a^ Regression	
	(n = 740)	(n = 426)	Cor (95%CI)	*p*	aOR (95%CI)	*p*
Smoked foods			1.81(1.48-2.21)	<0.001	2.44(1.89–3.13)	**<0.001**
Hardly ^b^	407(55.0%)	172(40.4%)	1		1	
Sometimes ^c^	310(41.9%)	213(50.0%)	1.63(1.27–2.09)	<0.001	2.14(1.57–2.91)	<0.001
Often ^d^	23(3.1%)	41(9.6%)	4.22(2.46–7.24)	<0.001	7.98(4.16–15.32)	<0.001
Barbecued foods			1.94(1.53–2.47)	<0.001	1.86(1.39–2.48)	**<0.001**
Hardly	558(75.4%)	260(61.0%)	1		1	
Sometimes	177(23.9%)	153(35.9%)	1.86(1.43–2.41)	<0.001	1.89(1.37–2.60)	<0.001
Often	5(0.7%)	13(3.1%)	5.58(1.97–15.82)	0.001	3.01(0.90–10.07)	0.073
Fried foods			1.55(1.27–1.89)	<0.001	1.93(1.51–2.46)	**<0.001**
Hardly	458(61.9%)	214(50.2%)	1		1	
Sometimes	253(34.2%)	177(41.5%)	1.50(1.16–1.92)	0.002	2.15(1.57–2.94)	<0.001
Often	29(3.9%)	35(8.2%)	2.58(1.54–4.34)	<0.001	3.02(1.62–5.60)	<0.001
Pickled vegetables			1.87(1.51–2.32)	<0.001	2.50(1.92–3.25)	**<0.001**
Hardly	448(60.5%)	184(43.2%)	1		1	
Sometimes	274(37.0%)	220(51.6%)	1.96(1.53–2.50)	<0.001	2.58(1.90–3.52)	<0.001
Often	18(2.4%)	22(5.2%)	2.98(1.56–5.68)	0.001	5.53(2.58–11.82)	<0.001
Fresh vegetables			0.89(0.52–1.52)	0.664	0.86(0.46–1.57)	0.615
Hardly	3(0.4%)	3(0.7%)	1		1	
Sometimes	21(2.8%)	12(2.8%)	0.57(0.10–3.29)	0.531	0.17(0.02–1.11)	0.064
Often	716(96.8%)	411(96.5%)	0.57(0.12–2.86)	0.498	0.24(0.04–1.27)	0.093
Fresh fruits			0.37(0.30–0.47)	<0.001	0.47(0.36–0.62)	**<0.001**
Hardly	14(1.9%)	81(19.0%)	1		1	
Sometimes	41(5.5%)	16(3.8%)	0.07(0.03–0.15)	<0.001	0.06(0.03–0.16)	<0.001
Often	685(92.6%)	329(77.2%)	0.08(0.05–0.15)	<0.001	0.12(0.06–0.24)	<0.001
Fresh meat			0.81(0.61–1.08)	0.155	1.08(0.77–1.54)	0.644
Hardly	21(2.8%)	12(2.8%)	1		1	
Sometimes	38(5.1%)	37(8.7%)	1.70(0.74–3.95)	0.214	1.41(0.52–3.84)	0.498
Often	681(92.0%)	377(88.5%)	0.97(0.47–1.99)	0.931	1.34(0.58–3.08)	0.493
Fish and shrimp			0.27(0.22–0.33)	<0.001	0.35(0.28–0.44)	**<0.001**
Hardly	29(3.9%)	91(21.4%)	1		1	
Sometimes	207(28.0%)	210(49.3%)	0.32(0.20–0.51)	<0.001	0.33(0.20–0.56)	<0.001
Often	504(68.1%)	125(29.3%)	0.08(0.05–0.12)	<0.001	0.12(0.07–0.20)	<0.001
Fresh eggs			0.40(0.33–0.49)	<0.001	0.56(0.45–0.71)	**<0.001**
Hardly	36(4.9%)	58(13.6%)	1		1	
Sometimes	86(11.6%)	127(29.8%)	0.92(0.56–1.51)	0.732	0.76(0.42–1.37)	0.355
Often	618(83.5%)	241(56.6%)	0.24(0.16–0.38)	<0.001	0.37(0.21–0.63)	<0.001
Beans			0.52(0.44–0.61)	<0.001	0.68(0.56–0.83)	**<0.001**
Hardly	107(14.5%)	107(25.1%)	1		1	
Sometimes	216(29.2%)	192(45.1%)	0.89(0.64–1.24)	0.486	1.13(0.76–1.69)	0.544
Often	417(56.4%)	127(29.8%)	0.30(0.22–0.42)	<0.001	0.52(0.35–0.79)	0.002
Milk products			0.51(0.44–0.59)	<0.001	0.67(0.56–0.80)	**<0.001**
Hardly	143(19.3%)	173(40.6%)	1		1	
Sometimes	150(20.3%)	109(25.6%)	0.60(0.43–0.84)	0.003	0.88(0.59–1.31)	0.533
Often	447(60.4%)	144(33.8%)	0.27(0.20–0.36)	<0.001	0.46(0.32–0.65)	<0.001

Abbreviations: VSD ventricular septal defect, cOR crude odds ratio, aOR adjusted odds ratio, CI confidence interval. ^a^ Adjusted for pre-pregnancy BMI, education level, consanguineous marriages, gestational diabetes mellitus, gestational hypertension, abnormal pregnancy history before this pregnancy, congenital malformations in family members, exposure to environmental pollutants, antibiotic use in early pregnancy, tobacco exposure in early pregnancy, alcohol exposure in early pregnancy, periconceptional folate use. ^b^ Hardly was defined as less than or equal to two times per week. ^c^ Sometimes was defined as three to five times per week. ^d^ Often was defined as more than or equal to six times per week.

**Table 3:** Genotypic frequencies of maternal *BHMT* polymorphisms and *P* values of HWE test.

SNPs	Location	Major Allele	Minor Allele	MAF	Group	Genotype Frequencies ^a^			*χ* ^2^	*p*
						AA	AB	BB		
rs3733890	Chr5: 79126136	G	A	0.3250	control	333(45.0%)	333(45.0%)	74(10.0%)	0.4865	0.4855
					case	162(38.0%)	216(50.7%)	48(11.3%)		
rs1316753	Chr5: 79235514	C	G	0.4338	control	248(33.5%)	342(46.2%)	150(20.3%)	2.5913	0.1075
					case	95(22.3%)	247(58.0%)	84(19.7%)		
rs567754	Chr5: 79120593	C	T	0.4628	control	203(27.4%)	389(52.6%)	148(20.0%)	2.4204	0.1198
					case	132(31.0%)	227(53.3%)	67(15.7%)		
rs1915706	Chr5: 79140388	T	C	0.2257	control	442(59.7%)	262(35.4%)	36(4.9%)	0.1261	0.7225
					case	223(52.3%)	176(41.3%)	27(6.3%)		

Abbreviations: *BHMT* betaine-homocysteine methyltransferase, HWE Hardy–Weinberg equilibrium, SNP single nucleotide polymorphism, MAF minimum allele frequency. ^a^ AA = homozygous wild—type; AB = heterozygous variant type; BB = homozygous variant type.

**Table 4:** Polymorphisms of maternal *BHMT* gene associated with risk of VSD in offspring based on logistic regression analysis.

SNPs	Univariate Logistic Reregression		Multivariate Logistic Regression ^a^		
	cOR (95%CI)	*p*	aOR (95%CI)	*p*	FDR_P
rs3733890					
GG	1		1		
GA	1.33(1.03–1.72)	0.026	1.28(0.94–1.73)	0.118	0.189
AA	1.33(0.89–2.01)	0.168	1.03(0.61–1.74)	0.918	0.918
Dominant model ^b^	1.33(1.04–1.70)	0.021	1.23(0.92–1.65)	0.163	0.217
Recessive model ^c^	1.14(0.78–1.68)	0.496	0.90(0.55–1.48)	0.681	0.904
Additive model ^d^	1.21(1.01–1.45)	0.038	1.11(0.88–1.39)	0.373	0.373
rs1316753					
CC	1		1		
CG	1.88(1.41–2.51)	<0.001	2.01(1.43–2.83)	<0.001	**<0.001**
GG	1.46(1.02–2.09)	0.037	1.55(1.00–2.40)	0.048	0.096
Dominant model	1.76(1.34–2.31)	<0.001	1.88(1.36–2.61)	<0.001	**<0.001**
Recessive model	0.97(0.72–1.30)	0.821	0.98(0.68–1.41)	0.904	0.904
Additive model	1.25(1.05–1.48)	0.012	1.30(1.06–1.60)	0.014	**0.028**
rs567754					
CC	1		1		
CT	0.90(0.68–1.18)	0.438	0.90(0.65–1.26)	0.555	0.634
TT	0.70(0.48–1.00)	0.050	0.78(0.51–1.19)	0.249	0.332
Dominant model	0.84(0.65–1.09)	0.197	0.87(0.64–1.20)	0.393	0.393
Recessive model	0.75(0.54–1.02)	0.071	0.83(0.57–1.20)	0.323	0.646
Additive model	0.84(0.71–1.01)	0.058	0.88(0.72–1.09)	0.255	0.340
rs1915706					
TT	1		1		
CT	1.33(1.04–1.71)	0.025	1.81(1.33–2.46)	<0.001	**<0.001**
CC	1.49(0.88–2.51)	0.138	2.05(1.10–3.82)	0.023	0.061
Dominant model	1.35(1.06–1.72)	0.014	1.84(1.37–2.48)	<0.001	**<0.001**
Recessive model	1.32(0.79–2.21)	0.285	1.60(0.88–2.94)	0.124	0.496
Additive model	1.28(1.05–1.56)	0.015	1.61(1.27–2.05)	<0.001	**<0.001**

Abbreviations: *BHMT* betaine-homocysteine methyltransferase, VSD ventricular septal defect, SNP single nucleotide polymorphism, cOR crude odds ratio, aOR adjusted odds ratio, CI confidence interval, FDR_P, false discovery rate P value. ^a^ Adjusted for pre-pregnancy BMI, education level, consanguineous marriages, gestational diabetes mellitus, gestational hypertension, abnormal pregnancy history before this pregnancy, congenital malformations in family members, exposure of environmental pollutants, antibiotic use in early pregnancy, tobacco exposure in early pregnancy, alcohol exposure in early pregnancy, periconceptional folate use. ^b^ The dominant model means heterozygote and mutant type homozygote vs. wild type homozygote. ^c^ The recessive model means mutant type homozygote vs. heterozygote and wild type homozygote. ^d^ The additive model means mutant type homozygote vs. heterozygote vs. mutant type homozygote.

**Table 5:** Interactions of polymorphisms of *BHMT* gene and maternal dietary habits based on multivariate logistic regression.

Dietary Habits ^a^	Interaction with rs1316753 ^b^			Interaction with rs1915706 ^b^		
	aOR (95%CI) ^c^	*p*	FDR_P	aOR (95%CI) ^c^	*p*	FDR_P
Smoked foods	0.52 (0.26–1.01)	0.055	0.165	0.62 (0.34–1.14)	0.122	0.305
Barbecued foods	1.24 (0.62–2.48)	0.548	0.616	1.33 (0.71–2.49)	0.377	0.610
Fried foods	1.40 (0.72–2.71)	0.316	0.406	1.19 (0.66–2.15)	0.570	0.634
Pickled vegetables	0.48 (0.24–0.95)	**0.034**	0.165	0.66 (0.36–1.19)	0.170	0.340
Fresh fruits	0.30 (0.05–1.68)	0.168	0.360	0.68 (0.18–2.58)	0.571	0.634
Fish and shrimp	0.85 (0.29–2.53)	0.776	0.776	0.66 (0.24–1.84)	0.427	0.610
Fresh eggs	2.37 (0.64–8.83)	0.200	0.360	0.39 (0.13–1.18)	0.095	0.305
Beans	0.40 (0.17–0.95)	**0.038**	0.165	0.33 (0.15–0.73)	**0.006**	**0.035**
Milk products	0.66 (0.32–1.38)	0.273	0.406	1.14 (0.60–2.19)	0.687	0.687

Abbreviations: *BHMT* betaine-homocysteine methyltransferase, aOR adjusted odds ratio, CI confidence interval, FDR_P, false discovery rate *p*-value. ^a^ Maternal dietary habits were classified as hardly and sometimes/often. ^b^ Single nucleotide polymorphisms were classified as wild-type and variant genotypes. ^c^ Adjusted for pre-pregnancy BMI, education level, consanguineous marriages, gestational diabetes mellitus, gestational hypertension, abnormal pregnancy history before this pregnancy, congenital malformations in family members, exposure to environmental pollutants, antibiotic use in early pregnancy, tobacco exposure in early pregnancy, alcohol exposure in early pregnancy, periconceptional folate use.

**Table 6:** Interaction of rs1915706 and maternal beans intake based on crossover analysis.

rs1915706 ^a^	Maternal Beans Intake ^b^	Cases	Controls	cOR(95%CI)	aOR(95%CI) ^c^
-	-	175 (41.1%)	356 (48.1%)	1	1
-	+	48 (11.3%)	86 (11.6%)	1.14 (0.76–1.69)	0.88 (0.54–1.42)
+	-	144 (33.8%)	277 (37.4%)	1.06 (0.81–1.39)	**1.52 (1.09–2.11)**
+	+	59 (13.8%)	21 (2.8%)	5.72 (3.36–9.71)	**4.00 (2.17–7.40)**

Abbreviations: cOR crude odds ratio, aOR adjusted odds ratio, CI confidence interval. ^a^ For rs1915706, ‘-’ means wild type, ‘+’ means variant genotype. ^b^ For maternal beans intake, ‘–’ means regular intake (namely, sometimes/often), ‘+’ means little intake (namely, hardly). ^c^ Adjusted for pre-pregnancy BMI, education level, consanguineous marriages, gestational diabetes mellitus, gestational hypertension, abnormal pregnancy history before this pregnancy, congenital malformations in family members, exposure to environmental pollutants, antibiotic use in early pregnancy, tobacco exposure in early pregnancy, alcohol exposure in early pregnancy, and periconceptional folate use.

## Data Availability

The data presented in this study are available on request from the corresponding author.
